# Predictive and prognostic biomarkers in cancer: towards the precision medicine era

**DOI:** 10.37349/etat.2024.00278

**Published:** 2024-11-20

**Authors:** Stefano Marletta, Antonio Rizzo, Graziana Spoto, Luca Falzone

**Affiliations:** University of Campania “Luigi Vanvitelli”, Italy; ^1^Division of Pathology, Humanitas Istituto Clinico Catanese, 95045 Catania, Italy; ^2^Department of Diagnostics and Public Health, Section of Pathology, University of Verona, 37134 Verona, Italy; ^3^Department of Biomedical and Biotechnological Sciences, University of Catania, 95123 Catania, Italy

Cancer research has made significant progress in recent years, shedding light on the molecular mechanisms that drive tumor development and progression. Advancements in this field have been related to identifying and applying predictive and prognostic biomarkers, which have emerged as key hallmarks in guiding therapeutic decisions and improving patients’ clinical outcomes. Notably, predictive biomarkers are pivotal to identifying patients who are likely to respond to specific treatments, thereby optimizing therapeutic efficacy and minimizing adverse effects [1]. In contrast, prognostic biomarkers provide valuable information on disease progression, offering insights into patient prognosis regardless of the treatment received [2].

## The significance of predictive and prognostic biomarkers

Among predictive biomarkers serving as critical indicators for tailoring treatment strategies, specific genetic, epigenetic, or protein-based markers associated with treatment response have emerged to allow clinicians to select the most appropriate therapies for individual patients. For instance, HER2 overexpression in breast cancer (BC) predicts the response to trastuzumab HER2-targeted therapy, significantly improving patient outcomes [3]. Similarly, mutations in the *EGFR* gene in non-small cell lung cancer (NSCLC) predict responsiveness to EGFR inhibitors like erlotinib and gefitinib, underscoring the transformative impact of predictive biomarkers in personalized medicine [4].

Prognostic biomarkers, on the other hand, offer insights into the likely course of the disease, independent of the chosen treatment. These biomarkers help stratify patients based on their risk of disease progression, enabling more informed decisions regarding surveillance and management. For example, the Oncotype DX test in BC assesses the expression of 21 genes to predict the likelihood of recurrence, guiding decisions on the necessity of adjuvant chemotherapy [5]. Such prognostic tools are invaluable in avoiding overtreatment and focusing resources on patients who will benefit the most.

## Technological advancements in biomarker discovery

Technological advancements in molecular biology and genomics have prompted the discovery and validation of predictive and prognostic biomarkers. Next-generation sequencing (NGS) has revolutionized this field of research, allowing comprehensive analysis of cancer genomes and the identification of novel biomarkers. Techniques such as whole-exome sequencing and RNA sequencing provide a detailed landscape of the genetic and transcriptomic alterations in tumors, facilitating the discovery of potential biomarkers [6].

Besides these novel high-throughput platforms, immunohistochemistry (IHC) remains a cornerstone in biomarker validation, offering a practical and reliable method for detecting protein expression in tissue samples. The development of multiplex IHC techniques has further enhanced the ability to analyze multiple biomarkers simultaneously, providing a more comprehensive understanding of tumor biology [7].

## The emerging role of liquid biopsy in cancer detection

Emerging technologies such as liquid biopsy and microfluidics for the detection of circulating tumor cells (CTCs) offer non-invasive approaches to biomarker discovery and disease monitoring. Liquid biopsy involves the analysis of tumor-derived components, such as circulating tumor DNA (ctDNA) or exosomes, in blood samples, as well as genetic and epigenetic hallmarks associated with specific tumors. This technique allows for real-time monitoring of tumor dynamics and the detection of minimal residual disease, offering a promising avenue for early diagnosis and treatment monitoring [8]. Due to the limitations of traditional biopsies, including their invasiveness and inability to reflect the tumor’s heterogeneity, liquid biopsy is revolutionizing cancer diagnosis as it can provide a more comprehensive view of the tumor’s evolving genomic landscape, capturing the emergence of drug-resistant clones and enabling timely adjustments to treatment strategies. Key applications of liquid biopsy are the detection of circulating *EGFR* mutations associated with NSCLC resistance or the detection of *ESR1* and *PIK3CA* mutations in BC predicting the response to hormonal treatments or the efficacy of targeted therapies [9, 10]. In addition, liquid biopsy can be used for the detection and quantification of ctDNA to assess the minimal residual disease following surgery or chemotherapy.

## The role of bioinformatics and artificial intelligence

Generating terabytes of omics data through high-throughput platforms has necessitated the development of bioinformatic solutions for the integrated analysis of complex biological data. Recently, these efforts have increasingly incorporated the aid of artificial intelligence (AI) in the search for biomarkers. The ability to analyze large-scale datasets from genomics, transcriptomics, proteomics, and metabolomics enables the identification of complex biomarker signatures that reflect the multifaceted nature of cancer. In addition, AI-driven algorithms can uncover patterns and correlations within these datasets, facilitating the discovery of novel biomarkers and enhancing the precision of existing ones [11].

For example, machine learning models can predict patient outcomes based on multi-omics data, providing a more accurate prognostic assessment. Additionally, AI can assist in interpreting imaging data from digital pathology investigations, correlating histochemical, radiographic, and molecular features to refine diagnosis and treatment planning [12].

## Epigenetic biomarkers and their clinical implications

Epigenetic modifications, such as DNA methylation and histone modifications, play a crucial role in cancer development and progression. Epigenetic biomarkers have emerged as valuable tools for early diagnosis, prognosis, and predicting treatment response. For instance, the methylation status of the *MGMT* gene promoter in glioblastoma is a predictive biomarker for responsiveness to alkylating agents like temozolomide [13].

Non-coding RNAs (ncRNAs) have also gained prominence as epigenetic biomarkers. These molecules, which include microRNAs (miRNAs) and long non-coding RNAs (lncRNAs), do not encode proteins but can regulate gene expression at various levels. Aberrant expression of specific ncRNAs has been linked to cancer, making them potential biomarkers for diagnosis, prognosis, and therapeutic targets. For instance, miR-21 is often upregulated in various cancers and is associated with poor prognosis and resistance to chemotherapy [14].

The analysis of epigenetic alterations in liquid biopsy samples offers a non-invasive approach to cancer detection and monitoring. Methylation-specific polymerase chain reaction (PCR) and bisulfite sequencing are among the techniques used to assess DNA methylation patterns, providing insights into tumor biology and therapeutic targets [15].

## Conclusions

Despite the advancements described above, the successful integration of predictive and prognostic biomarkers into clinical practice requires robust validation and standardization. Clinical trials play a pivotal role in demonstrating the utility of biomarkers in guiding treatment decisions and improving patient outcomes. Similarly, regulatory approval and the development of standardized assays are needed to ensure the reliability and reproducibility of biomarker tests. Therefore, collaborative efforts between researchers, clinicians, and industry stakeholders are essential for translating biomarker discoveries into clinical applications. Such interdisciplinary collaborations foster the exchange of knowledge and expertise, accelerating the implementation of precision medicine strategies in oncology.

Of note, the discovery of new biomarkers for the diagnosis of tumors is still limited by several challenges. Among these, intra- and inter-tumor heterogeneity and genetic variability can lead to inconsistent biomarker expression in different patients. To face this limitation, multi-biomarker panels or dynamic monitoring of biomarkers have been proposed to capture the evolving complexity of tumor biology with an inevitable increase in analysis costs. Other challenges are related to the lack of standard procedures for biomarker detection. Specifically, the variability in assay platforms, sample handling, and data interpretation often leads to conflicting results. Therefore, internationally recognized and validated guidelines are warranted to confirm the diagnostic accuracy of selected biomarkers.

In this complex scenario, some issues are related to the cost-benefit ratio related to the detection of molecular biomarkers using high-throughput platforms which limit the implementation of biomarker testing on a large scale. Therefore, in the near future, the development of specific assays and point-of-care platforms is mandatory to propose novel biomarkers for cancer screening. Finally, future research should prioritize real-world evidence to assess the long-term utility of biomarkers in routine practice, beyond the controlled environments of clinical trials.

Overall, the exploration and characterization of predictive and prognostic biomarkers represent a cornerstone of modern cancer research and clinical practice. Technological advancements in genomics, bioinformatics, and liquid biopsy have propelled the discovery of novel biomarkers, offering new avenues for personalized treatment and improved patient outcomes. The integration of these biomarkers into clinical practice heralds the era of precision medicine, where treatments are tailored to the unique molecular profile of each tumor, ultimately transforming the landscape of cancer diagnosis and management.

## Abbreviations

AI: artificial intelligence
BC: breast cancer
ctDNA: circulating tumor DNA
EGFR: epidermal growth factor receptor
HER2: human epidermal growth factor receptor 2
IHC: immunohistochemistry
ncRNAs: non-coding RNAs
NSCLC: non-small cell lung cancer

## Declarations

### Copyright

© The Author(s) 2024.
